# Supplementary material for: A fault-intrusion-tolerant system and deadline-aware algorithm for scheduling scientific workflow in the cloud
Source: PeerJ Comput Sci. 2021 Nov 2;7:e747. doi: 10.7717/peerj-cs.747 (PMC8576554; doi:10.7717/peerj-cs.747)
Supplement: Supplemental Information 6 [file peerj-cs-07-747-s006.zip › Myproject1.1/docs/org/cloudbus/cloudsim/VmScheduler.html]

VmScheduler


JavaScript is disabled on your browser.


- Overview
- Package
- Class
- Use
- Tree
- Deprecated
- Index
- Help

- Prev Class
- Next Class

- Frames
- No Frames

- All Classes

- Summary:
- Nested |
- Field |
- Constr |
- Method

- Detail:
- Field |
- Constr |
- Method


org.cloudbus.cloudsim

## Class VmScheduler

- java.lang.Object
- - org.cloudbus.cloudsim.VmScheduler

- Direct Known Subclasses:
  :   VmSchedulerSpaceShared, VmSchedulerTimeShared

  ---

    

  ```
  public abstract class VmScheduler
  extends java.lang.Object
  ```

  VmScheduler is an abstract class that represents the policy used by a VMM to share processing
  power among VMs running in a host.

  Since:
  :   CloudSim Toolkit 1.0

- - ### Constructor Summary

    Constructors

    Constructor and Description

    `VmScheduler(java.util.List<? extends Pe> pelist)`

    Creates a new HostAllocationPolicy.
  - ### Method Summary

    Methods

    | Modifier and Type | Method and Description |
    | --- | --- |
    | `abstract boolean` | `allocatePesForVm(Vm vm, java.util.List<java.lang.Double> mipsShare)` Allocates PEs for a VM. |
    | `void` | `deallocatePesForAllVms()` Releases PEs allocated to all the VMs. |
    | `abstract void` | `deallocatePesForVm(Vm vm)` Releases PEs allocated to a VM. |
    | `java.util.List<java.lang.Double>` | `getAllocatedMipsForVm(Vm vm)` Returns the MIPS share of each Pe that is allocated to a given VM. |
    | `double` | `getAvailableMips()` Gets the free mips. |
    | `double` | `getMaxAvailableMips()` Returns maximum available MIPS among all the PEs. |
    | `protected java.util.Map<java.lang.String,java.util.List<java.lang.Double>>` | `getMipsMap()` Gets the mips map. |
    | `double` | `getPeCapacity()` Returns PE capacity in MIPS. |
    | `<T extends Pe>  java.util.List<T>` | `getPeList()` Gets the vm list. |
    | `java.util.Map<java.lang.String,java.util.List<Pe>>` | `getPeMap()` Gets the pe map. |
    | `java.util.List<Pe>` | `getPesAllocatedForVM(Vm vm)` Gets the pes allocated for vm. |
    | `double` | `getTotalAllocatedMipsForVm(Vm vm)` Gets the total allocated MIPS for a VM over all the PEs. |
    | `java.util.List<java.lang.String>` | `getVmsMigratingIn()` Gets the vms migrating in. |
    | `java.util.List<java.lang.String>` | `getVmsMigratingOut()` Gets the vms in migration. |
    | `protected void` | `setAvailableMips(double availableMips)` Sets the free mips. |
    | `protected void` | `setMipsMap(java.util.Map<java.lang.String,java.util.List<java.lang.Double>> mipsMap)` Sets the mips map. |
    | `protected <T extends Pe>  void` | `setPeList(java.util.List<T> peList)` Sets the vm list. |
    | `protected void` | `setPeMap(java.util.Map<java.lang.String,java.util.List<Pe>> peMap)` Sets the pe map. |
    | `protected void` | `setVmsMigratingIn(java.util.List<java.lang.String> vmsMigratingIn)` Sets the vms migrating in. |
    | `protected void` | `setVmsMigratingOut(java.util.List<java.lang.String> vmsInMigration)` Sets the vms in migration. |

    - ### Methods inherited from class java.lang.Object

      `clone, equals, finalize, getClass, hashCode, notify, notifyAll, toString, wait, wait, wait`

- - ### Constructor Detail


    - #### VmScheduler

      ```
      public VmScheduler(java.util.List<? extends Pe> pelist)
      ```

      Creates a new HostAllocationPolicy.

      Parameters:
      :   `pelist` - the pelist
  - ### Method Detail


    - #### allocatePesForVm

      ```
      public abstract boolean allocatePesForVm(Vm vm,
                             java.util.List<java.lang.Double> mipsShare)
      ```

      Allocates PEs for a VM.

      Parameters:
      :   `vm` - the vm
      :   `mipsShare` - the mips share

      Returns:
      :   $true if this policy allows a new VM in the host, $false otherwise


    - #### deallocatePesForVm

      ```
      public abstract void deallocatePesForVm(Vm vm)
      ```

      Releases PEs allocated to a VM.

      Parameters:
      :   `vm` - the vm


    - #### deallocatePesForAllVms

      ```
      public void deallocatePesForAllVms()
      ```

      Releases PEs allocated to all the VMs.


    - #### getPesAllocatedForVM

      ```
      public java.util.List<Pe> getPesAllocatedForVM(Vm vm)
      ```

      Gets the pes allocated for vm.

      Parameters:
      :   `vm` - the vm

      Returns:
      :   the pes allocated for vm


    - #### getAllocatedMipsForVm

      ```
      public java.util.List<java.lang.Double> getAllocatedMipsForVm(Vm vm)
      ```

      Returns the MIPS share of each Pe that is allocated to a given VM.

      Parameters:
      :   `vm` - the vm

      Returns:
      :   an array containing the amount of MIPS of each pe that is available to the VM


    - #### getTotalAllocatedMipsForVm

      ```
      public double getTotalAllocatedMipsForVm(Vm vm)
      ```

      Gets the total allocated MIPS for a VM over all the PEs.

      Parameters:
      :   `vm` - the vm

      Returns:
      :   the allocated mips for vm


    - #### getMaxAvailableMips

      ```
      public double getMaxAvailableMips()
      ```

      Returns maximum available MIPS among all the PEs.

      Returns:
      :   max mips


    - #### getPeCapacity

      ```
      public double getPeCapacity()
      ```

      Returns PE capacity in MIPS.

      Returns:
      :   mips


    - #### getPeList

      ```
      public <T extends Pe> java.util.List<T> getPeList()
      ```

      Gets the vm list.

      Type Parameters:
      :   `T` - the generic type

      Returns:
      :   the vm list


    - #### setPeList

      ```
      protected <T extends Pe> void setPeList(java.util.List<T> peList)
      ```

      Sets the vm list.

      Type Parameters:
      :   `T` - the generic type

      Parameters:
      :   `peList` - the pe list


    - #### getMipsMap

      ```
      protected java.util.Map<java.lang.String,java.util.List<java.lang.Double>> getMipsMap()
      ```

      Gets the mips map.

      Returns:
      :   the mips map


    - #### setMipsMap

      ```
      protected void setMipsMap(java.util.Map<java.lang.String,java.util.List<java.lang.Double>> mipsMap)
      ```

      Sets the mips map.

      Parameters:
      :   `mipsMap` - the mips map


    - #### getAvailableMips

      ```
      public double getAvailableMips()
      ```

      Gets the free mips.

      Returns:
      :   the free mips


    - #### setAvailableMips

      ```
      protected void setAvailableMips(double availableMips)
      ```

      Sets the free mips.

      Parameters:
      :   `availableMips` - the new free mips


    - #### getVmsMigratingOut

      ```
      public java.util.List<java.lang.String> getVmsMigratingOut()
      ```

      Gets the vms in migration.

      Returns:
      :   the vms in migration


    - #### setVmsMigratingOut

      ```
      protected void setVmsMigratingOut(java.util.List<java.lang.String> vmsInMigration)
      ```

      Sets the vms in migration.

      Parameters:
      :   `vmsInMigration` - the new vms migrating out


    - #### getVmsMigratingIn

      ```
      public java.util.List<java.lang.String> getVmsMigratingIn()
      ```

      Gets the vms migrating in.

      Returns:
      :   the vms migrating in


    - #### setVmsMigratingIn

      ```
      protected void setVmsMigratingIn(java.util.List<java.lang.String> vmsMigratingIn)
      ```

      Sets the vms migrating in.

      Parameters:
      :   `vmsMigratingIn` - the new vms migrating in


    - #### getPeMap

      ```
      public java.util.Map<java.lang.String,java.util.List<Pe>> getPeMap()
      ```

      Gets the pe map.

      Returns:
      :   the pe map


    - #### setPeMap

      ```
      protected void setPeMap(java.util.Map<java.lang.String,java.util.List<Pe>> peMap)
      ```

      Sets the pe map.

      Parameters:
      :   `peMap` - the pe map


- Overview
- Package
- Class
- Use
- Tree
- Deprecated
- Index
- Help

- Prev Class
- Next Class

- Frames
- No Frames

- All Classes

- Summary:
- Nested |
- Field |
- Constr |
- Method

- Detail:
- Field |
- Constr |
- Method
